# The genome sequence of the Large Sharp-tail Bee, *Coelioxys conoideus *(Illiger,1806)

**DOI:** 10.12688/wellcomeopenres.19507.1

**Published:** 2023-06-21

**Authors:** Susan C Taylor, Sally Luker, William L S Hawkes, Natural History Museum Genome Acquisition Lab, Darwin Tree of Life Barcoding collective, Wellcome Sanger Institute Tree of Life programme, Wellcome Sanger Institute Scientific Operations: DNA Pipelines collective, Tree of Life Core Informatics collective, Darwin Tree of Life Consortium

**Affiliations:** 1 Dipterists Forum, Northchurch, England, UK; 2 University of Exeter, Exeter, England, UK

**Keywords:** Coelioxys conoideus, Large Sharp-tail Bee, genome sequence, chromosomal, Hymenoptera

## Abstract

We present a genome assembly from an individual female *Coelioxys conoideus* (the Large Sharp-tail Bee; Arthropoda; Insecta; Hymenoptera; Megachilidae). The genome sequence is 417.6 megabases in span. Most of the assembly is scaffolded into 12 chromosomal pseudomolecules. The mitochondrial genome has also been assembled and is 20.8 kilobases in length.

## Species taxonomy

Eukaryota; Metazoa; Eumetazoa; Bilateria; Protostomia; Ecdysozoa; Panarthropoda; Arthropoda; Mandibulata; Pancrustacea; Hexapoda; Insecta; Dicondylia; Pterygota; Neoptera; Endopterygota; Hymenoptera; Apocrita; Aculeata; Apoidea; Anthophila; Megachilidae; Megachilinae; Megachilini; *Coelioxys*, *Coelioxys conoideus* (Illiger,1806) (NCBI:txid2922063).

## Background

*Coelioxys conoidea* (Illiger, 1806) is a medium-sized bee (11–15 mm), the largest of the six UK species of sharp-tailed bees (Tribe: Megachilini), with a forewing length of 7–9 mm ( Falk & Lewington, 2019). Both sexes have bright white hairs on the sides of the tergites and sternites, with interrupted hair bands present on tergites T2–T5 and sternites S2–S4. White hairs are present also on the face and sides of the thorax. The female has a cone-shaped abdomen, typical of the sharp tailed bees, with the final (6th) sternite being distinctly boat-shaped. The male has a broader abdomen, the final sternite bearing a pair of prominent, blunt teeth. For identification purposes, pictorial comparisons of UK *Coelioxys* spp. can be found in ( Rowson & Pavett, 2008).

Sharp-tailed bees are kleptoparasites (cuckoos) of leaf-cutter bees ( *Megachile* spp.) or flower bees ( *Anthophor*a spp.), and *C. conoidea* are specifically kleptoparasites of *Megachile maritima* (Kirby, 1802). A female uses her pointed abdomen to pierce and oviposit into the cell of the host. The larva then hatches before that of the host, whereupon it consumes the food stores collected for the latter, while destroying the host egg or newly-hatched larva ( Bohart, 1970; Rowson & Pavett, 2008). In Europe, *C. conoidea* is known to be a kleptoparasite of *M. lagopoda,* which does not occur in the UK.

*Coelioxys conoidea* is a univoltine species, flying from June to August. The distribution in the UK follows the distribution of *Megachile maritima*, being mainly coastal, especially where sand dunes are present, while inland, it can be found on sandy heaths and occasionally on chalk. Adults are known to visit a wide range of flowering plants, including sea holly ( *Eryngium maritimum*, brambles ( *Rubus* spp.), knapweeds ( *Centaurea* spp.) and ragworts ( *Jacobaea* and *Senecio* spp.) ( BWARS, 2023; Falk & Lewington, 2019). In the UK, *C. conoidea* is mainly restricted to southern England and Wales, including the Channel Islands ( BWARS, 2023; Else *et al.*, 2016; Falk & Lewington, 2019; NBN Atlas Partnership, 2023), although recent records from Nottinghamshire, Yorkshire and South Lancashire ( BWARS, 2023; Falk & Lewington, 2019; NBN Atlas Partnership, 2023) suggest a possible range expansion. It is not regarded as scarce and can be locally common ( Falk & Lewington, 2019).

On the continent *C. conoidea* is found throughout much of Europe and the Middle East ( GBIF Secretariat, 2022). While it is listed as a species of Least Concern on the IUCN Red List Category (Europe) ( Nieto *et al.*, 2014; Ortiz Sánchez, 2014), in a more recent assessment, the Swedish Red List 2020 now lists *C. conoidea* as Critically Endangered ( SLU Artdatabanken, 2020) due to a declining population.

The genome of *Coelioxys conoidea* was sequenced as part of the Darwin Tree of Life Project, a collaborative effort to sequence all named eukaryotic species in the Atlantic Archipelago of Britain and Ireland. Here we present a chromosomally complete genome sequence for *Coelioxys conoidea*, based on one female specimen from Penhale Dunes, Cornwall.

## Genome sequence report

**Figure 1.**
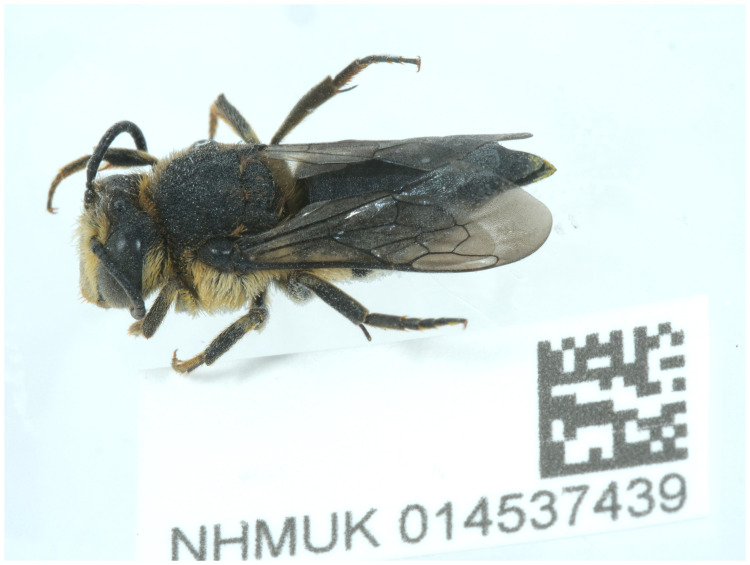
Photograph of the *Coelioxys conoideus* (iyCoeConi1) specimen used for genome sequencing.

The genome was sequenced from one female *Coelioxys conoideus* ( Figure 1) collected from Penhale Dunes, Cornwall (50.37, –5.12). A total of 41-fold coverage in Pacific Biosciences single-molecule HiFi long reads was generated. Primary assembly contigs were scaffolded with chromosome conformation Hi-C data. Manual assembly curation corrected 13 missing joins or mis-joins, reducing the scaffold number by 9.38%, and increasing the scaffold N50 by 11.35%.

**Figure 2.**
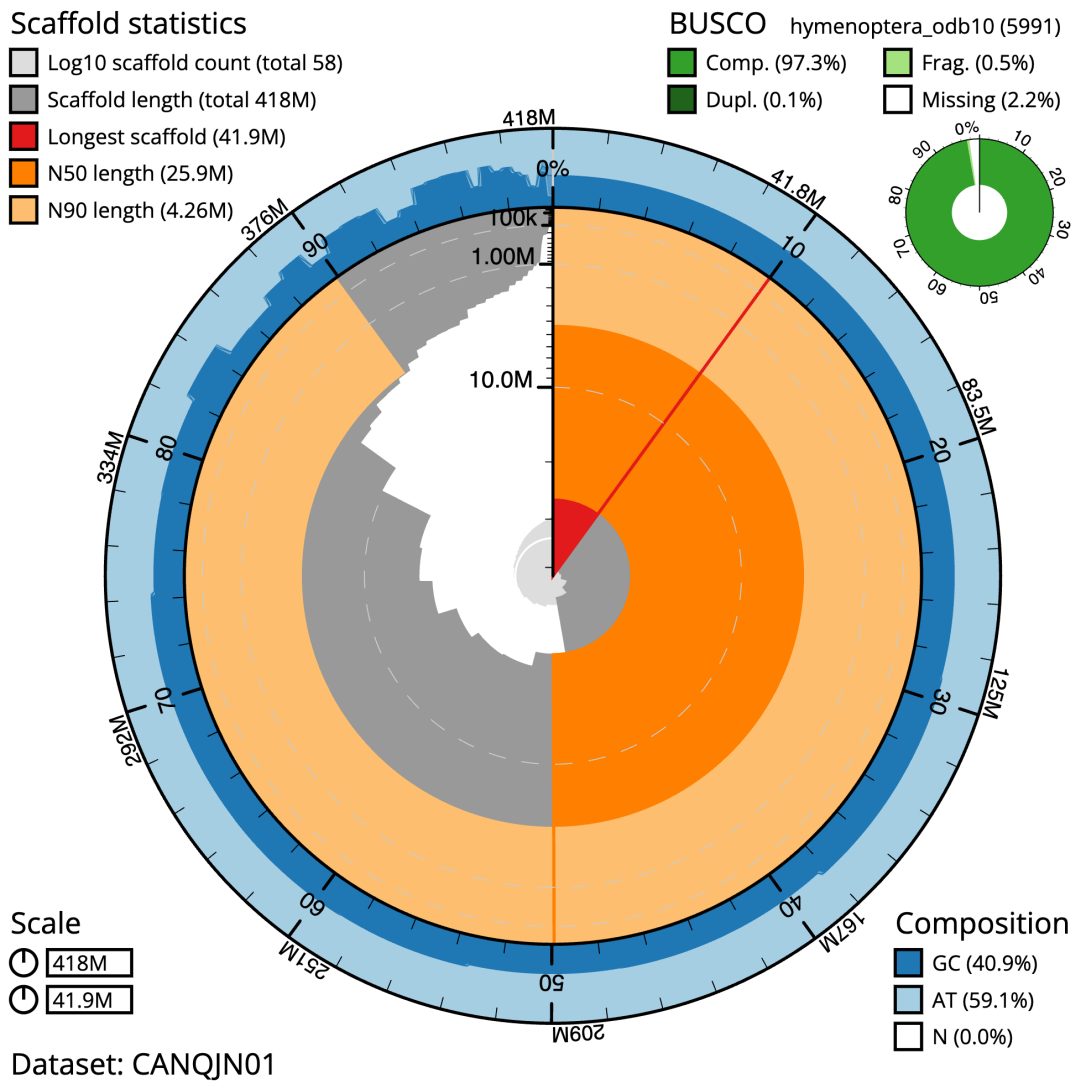
Genome assembly of *Coelioxys conoideus*, iyCoeConi1.1: metrics. The BlobToolKit Snailplot shows N50 metrics and BUSCO gene completeness. The main plot is divided into 1,000 size-ordered bins around the circumference with each bin representing 0.1% of the 417,592,752 bp assembly. The distribution of scaffold lengths is shown in dark grey with the plot radius scaled to the longest scaffold present in the assembly (41,884,646 bp, shown in red). Orange and pale-orange arcs show the N50 and N90 scaffold lengths (25,869,242 and 4,259,916 bp), respectively. The pale grey spiral shows the cumulative scaffold count on a log scale with white scale lines showing successive orders of magnitude. The blue and pale-blue area around the outside of the plot shows the distribution of GC, AT and N percentages in the same bins as the inner plot. A summary of complete, fragmented, duplicated and missing BUSCO genes in the hymenoptera_odb10 set is shown in the top right. An interactive version of this figure is available at https://blobtoolkit.genomehubs.org/view/iyCoeConi1.1/dataset/CANQJN01/snail.

The final assembly has a total length of 417.6 Mb in 58 sequence scaffolds with a scaffold N50 of 25.9 Mb ( Table 1). Most (82.24%) of the assembly sequence was assigned to 12 chromosomal-level scaffolds. Chromosome-scale scaffolds confirmed by the Hi-C data are named in order of size ( Figure 2– Figure 5; Table 2). While not fully phased, the assembly deposited is of one haplotype. Contigs corresponding to the second haplotype have also been deposited. The mitochondrial genome was also assembled and can be found as a contig within the multifasta file of the genome submission.

**Table 1.** Genome data for *Coelioxys conoideus*, iyCoeConi1.1.

Project accession data		
Assembly identifier	iyCoeConi1.1	
Species	*Coelioxys conoideus*	
Specimen	iyCoeConi1	
NCBI taxonomy ID	2922063	
BioProject	PRJEB55747	
BioSample ID	SAMEA14448263	
Isolate information	iyCoeConi1; abdomen (DNA sequencing), head and thorax (Hi-C scaffolding)	
Assembly metrics *		*Benchmark*
Consensus quality (QV)	66.1	*≥ 50*
*k*-mer completeness	100%	*≥ 95%*
BUSCO **	C:97.3%[S:97.2%,D:0.1%], F:0.5%,M:2.2%,n:5,991	*C ≥ 95%*
Percentage of assembly mapped to chromosomes	82.24%	*≥ 95%*
Sex chromosomes	-	*localised homologous pairs*
Organelles	Mitochondrial genomes assembled	*complete single alleles*
Raw data accessions		
PacificBiosciences SEQUEL II	ERR10168733	
Hi-C Illumina	ERR10149566	
Genome assembly		
Assembly accession	GCA_947623535.1	
*Accession of alternate haplotype*	GCA_947623525.1	
Span (Mb)	417.6	
Number of contigs	155	
Contig N50 length (Mb)	4.9	
Number of scaffolds	58	
Scaffold N50 length (Mb)	25.9	
Longest scaffold (Mb)	41.9	

* Assembly metric benchmarks are adapted from column VGP-2020 of “Table 1: Proposed standards and metrics for defining genome assembly quality” from ( Rhie *et al.*, 2021). ** BUSCO scores based on the hymenoptera_odb10 BUSCO set using v5.3.2. C = complete [S = single copy, D = duplicated], F = fragmented, M = missing, n = number of orthologues in comparison. A full set of BUSCO scores is available at https://blobtoolkit.genomehubs.org/view/iyCoeConi1.1/dataset/CANQJN01/busco.

**Figure 3.**
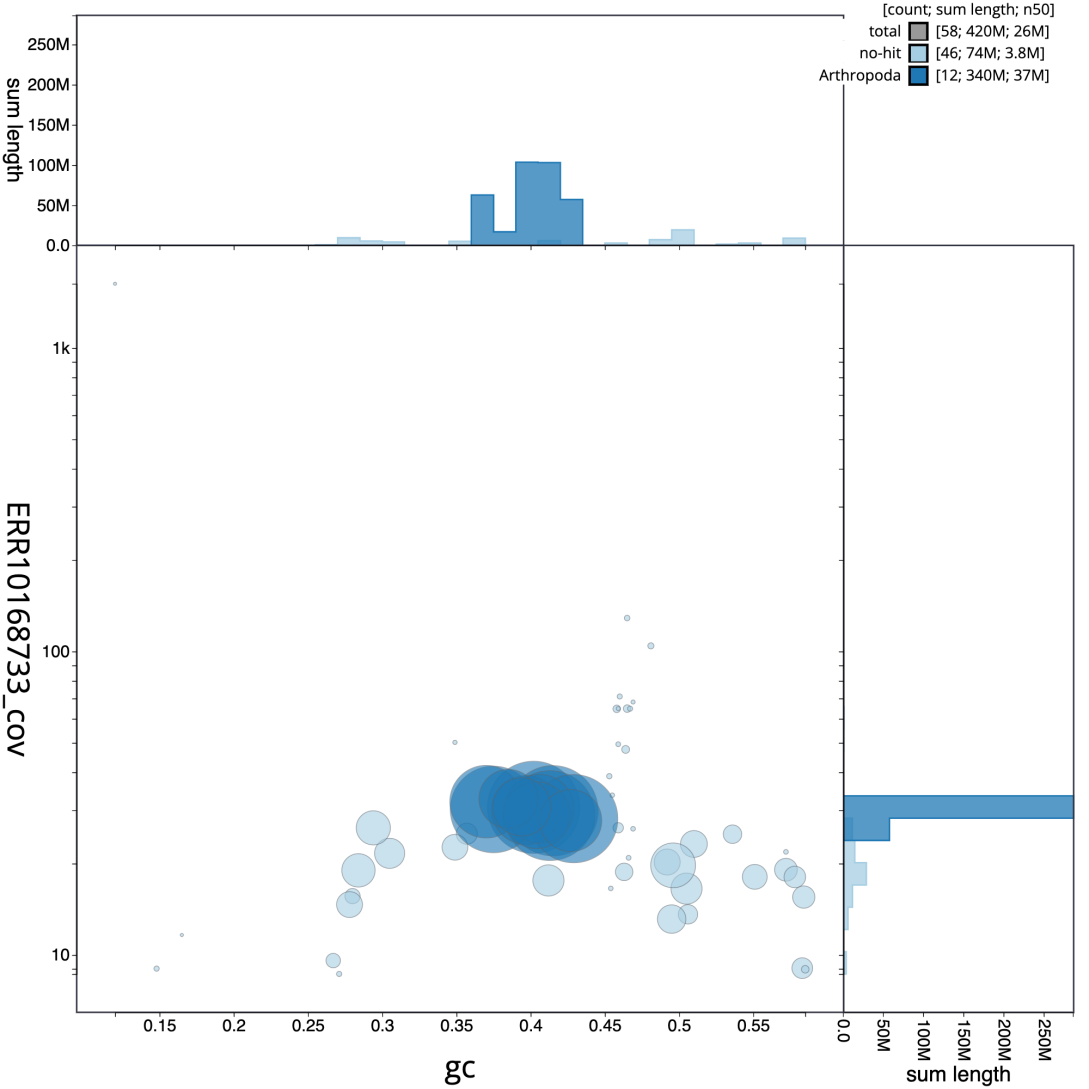
Genome assembly of *Coelioxys conoideus*, iyCoeConi1.1: BlobToolKit GC-coverage plot. Scaffolds are coloured by phylum. Circles are sized in proportion to scaffold length. Histograms show the distribution of scaffold length sum along each axis. An interactive version of this figure is available at https://blobtoolkit.genomehubs.org/view/iyCoeConi1.1/dataset/CANQJN01/blob.

**Figure 4.**
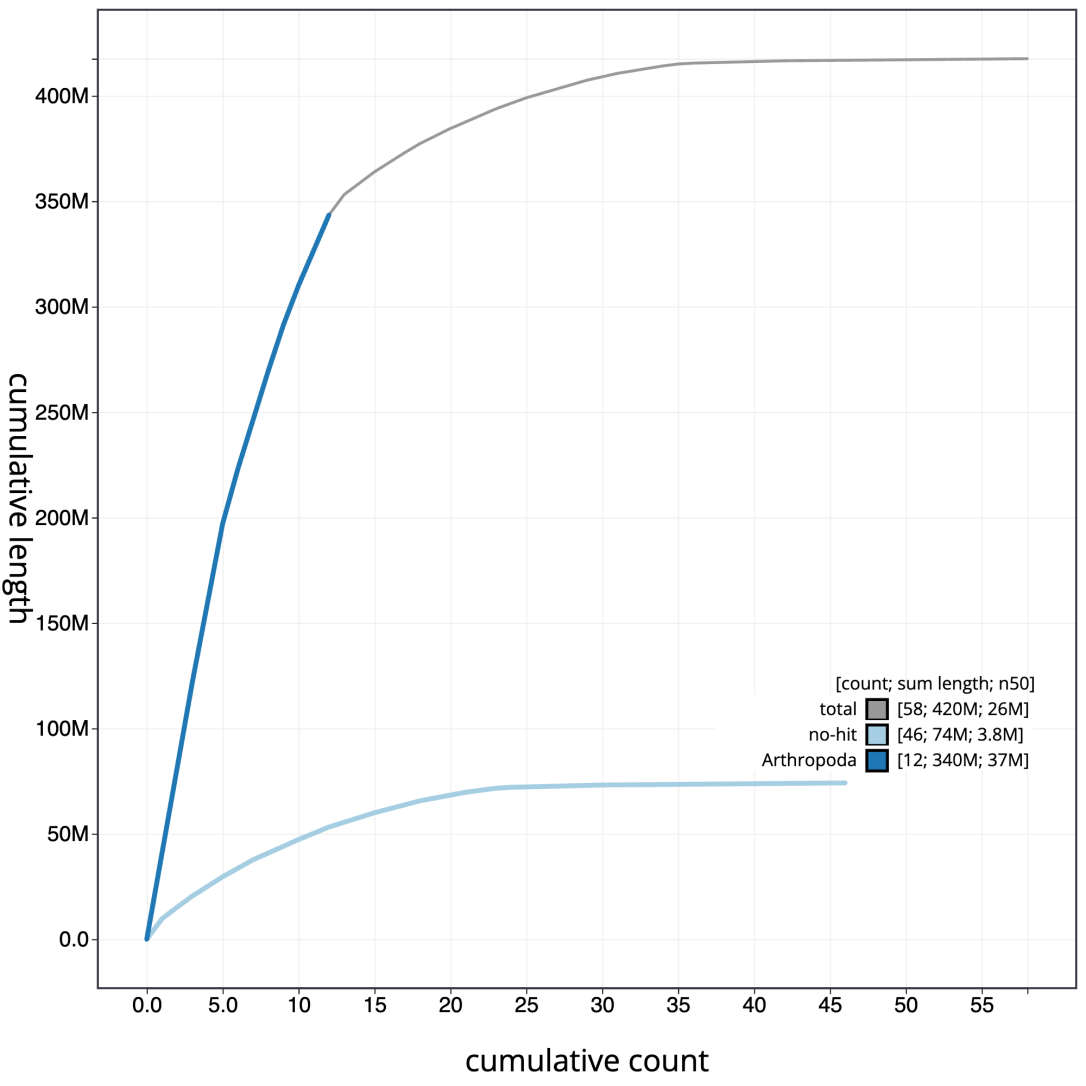
Genome assembly of *Coelioxys conoideus*, iyCoeConi1.1: BlobToolKit cumulative sequence plot. The grey line shows cumulative length for all scaffolds. Coloured lines show cumulative lengths of scaffolds assigned to each phylum using the buscogenes taxrule. An interactive version of this figure is available at https://blobtoolkit.genomehubs.org/view/iyCoeConi1.1/dataset/CANQJN01/cumulative.

**Figure 5.**
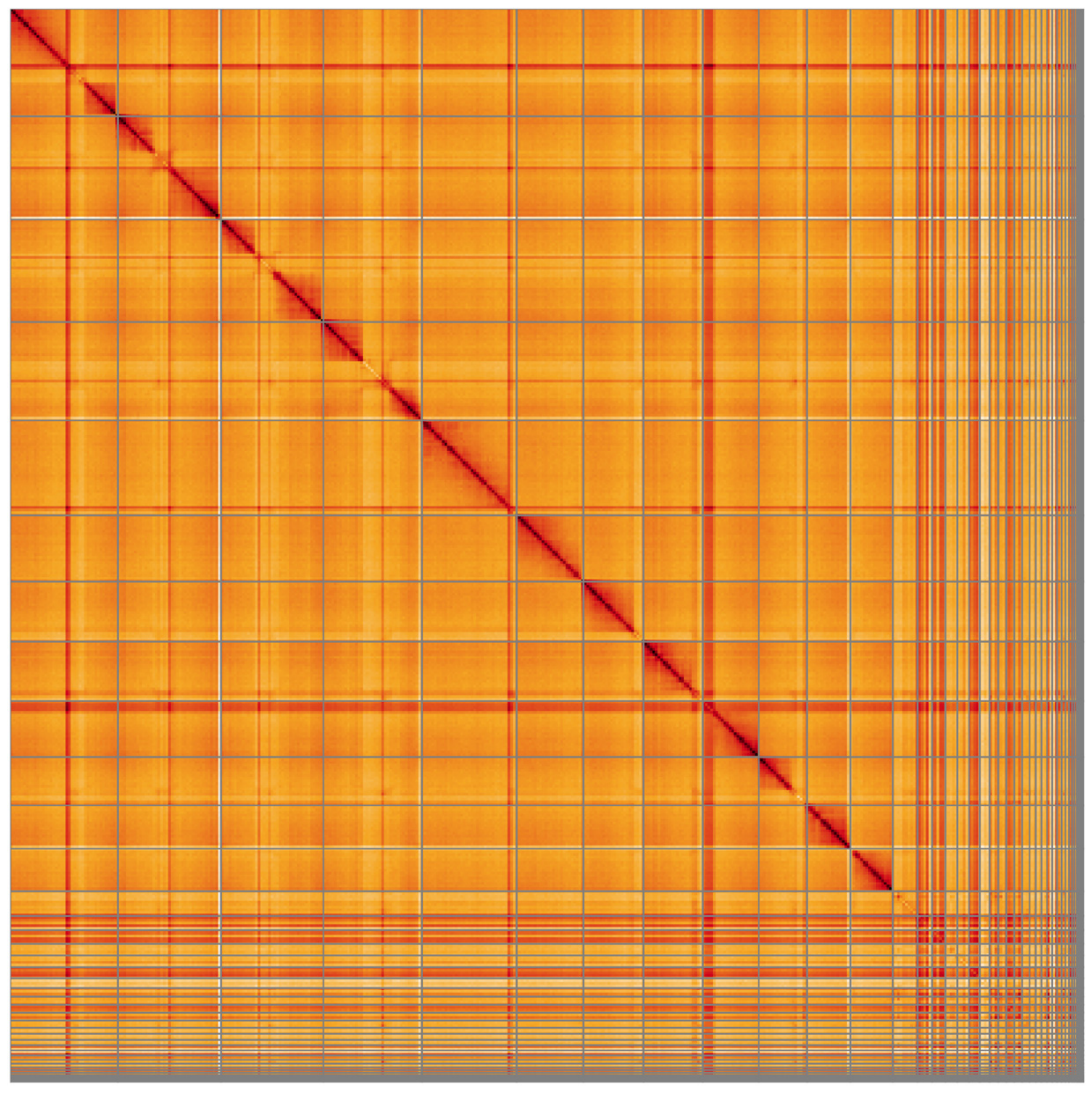
Genome assembly of *Coelioxys conoideus*, iyCoeConi1.1: Hi-C contact map of the iyCoeConi1.1 assembly, visualised using HiGlass. Chromosomes are shown in order of size from left to right and top to bottom. An interactive version of this figure may be viewed at https://genome-note-higlass.tol.sanger.ac.uk/l/?d=HpLT1q_ASdqw_NYwF8ARig.

**Table 2.** Chromosomal pseudomolecules in the genome assembly of *Coelioxys conoideus*, iyCoeConi1.

INSDC accession	Name	Length (Mb)	GC%
OX392449.1	1	41.88	40
OX392450.1	2	40.16	41.5
OX392451.1	3	39.81	41.5
OX392452.1	4	38.37	43
OX392453.1	5	36.91	37.5
OX392454.1	6	25.87	37
OX392455.1	7	23.35	39.5
OX392456.1	8	23.12	40.5
OX392457.1	9	21.8	40.5
OX392458.1	10	18.8	42.5
OX392459.1	11	16.89	38.5
OX392460.1	12	16.49	39.5
OX392461.1	MT	0.02	12

The estimated Quality Value (QV) of the final assembly is 66.1 with *k*-mer completeness of 100%, and the assembly has a BUSCO v5.3.2 completeness of 97.3% (single = 97.2%, duplicated = 0.1%), using the hymenoptera_odb10 reference set ( *n* = 5,991).

Metadata for specimens, spectral estimates, sequencing runs, contaminants and pre-curation assembly statistics can be found at https://links.tol.sanger.ac.uk/species/2922063.

## Methods

### Sample acquisition and nucleic acid extraction

A female *Coelioxys conoideus* (iyCoeConi1) was collected from Penhale Dunes, Cornwall, UK (latitude 50.17, longitude –5.12) on 2021-06-30. The specimen was collected by Sue Taylor (Dipterists’ Forum) and Sally Luker (University of Exeter), using an aerial net. The specimen was identified by Will Hawkes (University of Exeter) and Sue Taylor, and was snap-frozen on dry ice.

DNA was extracted at the Tree of Life laboratory, Wellcome Sanger Institute (WSI). The iyCoeConi1 sample was weighed and dissected on dry ice with tissue set aside for Hi-C sequencing. Abdomen tissue was disrupted using a Nippi Powermasher fitted with a BioMasher pestle. High molecular weight (HMW) DNA was extracted using the Qiagen MagAttract HMW DNA extraction kit. HMW DNA was sheared into an average fragment size of 12–20 kb in a Megaruptor 3 system with speed setting 30. Sheared DNA was purified by solid-phase reversible immobilisation using AMPure PB beads with a 1.8X ratio of beads to sample to remove the shorter fragments and concentrate the DNA sample. The concentration of the sheared and purified DNA was assessed using a Nanodrop spectrophotometer and Qubit Fluorometer and Qubit dsDNA High Sensitivity Assay kit. Fragment size distribution was evaluated by running the sample on the FemtoPulse system.

### Sequencing

Pacific Biosciences HiFi circular consensus DNA sequencing libraries were constructed according to the manufacturers’ instructions. DNA sequencing was performed by the Scientific Operations core at the WSI on Pacific Biosciences SEQUEL II (HiFi) instrument. Hi-C data were also generated from head and thorax tissue of iyCoeConi1 using the Arimav2 kit and sequenced on the Illumina NovaSeq 6000 instrument.

### Genome assembly, curation and evaluation

Assembly was carried out with Hifiasm ( Cheng *et al.*, 2021) and haplotypic duplication was identified and removed with purge_dups ( Guan *et al.*, 2020). The assembly was then scaffolded with Hi-C data ( Rao *et al.*, 2014) using YaHS ( Zhou *et al.*, 2023). The assembly was checked for as described previously ( Howe *et al.*, 2021). Manual curation was performed using HiGlass ( Kerpedjiev *et al.*, 2018) and Pretext ( Harry, 2022). The mitochondrial genome was assembled using MitoHiFi ( Uliano-Silva *et al.*, 2022), which runs MitoFinder ( Allio *et al.*, 2020) or MITOS ( Bernt *et al.*, 2013) and uses these annotations to select the final mitochondrial contig and to ensure the general quality of the sequence.

A Hi-C map for the final assembly was produced using bwa-mem2 ( Vasimuddin *et al.*, 2019) in the Cooler file format ( Abdennur & Mirny, 2020). To assess the assembly metrics, the *k*-mer completeness and QV consensus quality values were calculated in Merqury ( Rhie *et al.*, 2020). This work was done using Nextflow ( Di Tommaso *et al.*, 2017) DSL2 pipelines “sanger-tol/readmapping” ( Surana *et al.*, 2023a) and “sanger-tol/genomenote” ( Surana *et al.*, 2023b). The genome was analysed within the BlobToolKit environment ( Challis *et al.*, 2020) and BUSCO scores ( Manni *et al.*, 2021; Simão *et al.*, 2015) were calculated.

Table 3 contains a list of relevant software tool versions and sources.

**Table 3.** Software tools: versions and sources.

Software tool	Version	Source
BlobToolKit	4.0.7	https://github.com/blobtoolkit/blobtoolkit
BUSCO	5.3.2	https://gitlab.com/ezlab/busco
Hifiasm	0.16.1-r375	https://github.com/chhylp123/hifiasm
HiGlass	1.11.6	https://github.com/higlass/higlass
Merqury	MerquryFK	https://github.com/thegenemyers/MERQURY.FK
MitoHiFi	2	https://github.com/marcelauliano/MitoHiFi
PretextView	0.2	https://github.com/wtsi-hpag/PretextView
purge_dups	1.2.3	https://github.com/dfguan/purge_dups
sanger-tol/genomenote	v1.0	https://github.com/sanger-tol/genomenote
sanger-tol/readmapping	1.1.0	https://github.com/sanger-tol/readmapping/tree/1.1.0
YaHS	yahs-1.1.91eebc2	https://github.com/c-zhou/yahs

### Wellcome Sanger Institute – Legal and Governance

The materials that have contributed to this genome note have been supplied by a Darwin Tree of Life Partner.

The submission of materials by a Darwin Tree of Life Partner is subject to the **‘Darwin Tree of Life Project Sampling Code of Practice’**, which can be found in full on the Darwin Tree of Life website here. By agreeing with and signing up to the Sampling Code of Practice, the Darwin Tree of Life Partner agrees they will meet the legal and ethical requirements and standards set out within this document in respect of all samples acquired for, and supplied to, the Darwin Tree of Life Project.

Further, the Wellcome Sanger Institute employs a process whereby due diligence is carried out proportionate to the nature of the materials themselves, and the circumstances under which they have been/are to be collected and provided for use. The purpose of this is to address and mitigate any potential legal and/or ethical implications of receipt and use of the materials as part of the research project, and to ensure that in doing so we align with best practice wherever possible.

The overarching areas of consideration are:

- Ethical review of provenance and sourcing of the material
- Legality of collection, transfer and use (national and international)

Each transfer of samples is further undertaken according to a Research Collaboration Agreement or Material Transfer Agreement entered into by the Darwin Tree of Life Partner, Genome Research Limited (operating as the Wellcome Sanger Institute), and in some circumstances other Darwin Tree of Life collaborators.

## Data Availability

European Nucleotide Archive: *Coelioxys conoideus*. Accession number PRJEB55747; https://identifiers.org/ena.embl/PRJEB55747. ( Wellcome Sanger Institute, 2023) The genome sequence is released openly for reuse. The *Coelioxys conoideus* genome sequencing initiative is part of the Darwin Tree of Life (DToL) project. All raw sequence data and the assembly have been deposited in INSDC databases. The genome will be annotated using available RNA-Seq data and presented through the Ensembl pipeline at the European Bioinformatics Institute. Raw data and assembly accession identifiers are reported in Table 1.

## Author information

Members of the Natural History Museum Genome Acquisition Lab are listed here: https://doi.org/10.5281/zenodo.4790042.

Members of the Darwin Tree of Life Barcoding collective are listed here: https://doi.org/10.5281/zenodo.4893703.

Members of the Wellcome Sanger Institute Tree of Life programme are listed here: https://doi.org/10.5281/zenodo.4783585.

Members of Wellcome Sanger Institute Scientific Operations: DNA Pipelines collective are listed here: https://doi.org/10.5281/zenodo.4790455.

Members of the Tree of Life Core Informatics collective are listed here: https://doi.org/10.5281/zenodo.5013541.

Members of the Darwin Tree of Life Consortium are listed here: https://doi.org/10.5281/zenodo.4783558.
