# Supplementary material for: The genome sequence of the Large Sharp-tail Bee, Coelioxys conoideus (Illiger,1806)
Source: Wellcome Open Res. 2023 Jun 21;8:259. [Version 1] doi: 10.12688/wellcomeopenres.19507.1 (PMC11208850; doi:10.12688/wellcomeopenres.19507.1)
Supplement: Peer review history

# Reviewer report: Reviewer response for version 1

**DOI:** 10.21956/wellcomeopenres.21609.r87080

**Published:** 2024-06-26

**Authors:** Hongmei Li-Byarlay, Patricia Azevedo

**Affiliations:** 1 Agriculture Research and Development Program, Central State University, Wilberforce, Ohio, USA; 2 Campinas State University, Campinas, Brazil

This data note titled "The genome sequence of the large sharp-tail bee, Coelioxys conideus (Illiger, 1806)" is in line with the objectives proposed by the Darwin Tree of Life Consortium and consequently by the Earth Biogenome Project, which is to improve conservation strategies, preserve biodiversity and maintain ecosystem services vital to human well-being.

 For the biological facts, we wonder if the authors can add the info on how long the female bee can live, and what type of social status this species has if the info is available.

 The identification and sequencing of the samples were carried out according to the specifications of this project.

 An important information that would be welcome in the study is confirmation or evidence that the number of chromosomes of the species matches that described.

 Do reference genomes exist for other species in the genus? How many chromosomes did they have? Is synteny analysis possible?

 The figures could be improved by editing the x-axis of fig 5 if it is possible. So readers have some idea about this graph.

Are sufficient details of methods and materials provided to allow replication by others?

Yes

Is the rationale for creating the dataset(s) clearly described?

Yes

Are the datasets clearly presented in a useable and accessible format?

Yes

Are the protocols appropriate and is the work technically sound?

Yes

Reviewer Expertise:

bee genomics, bee genetics, entomology, ecological genomics; parasitology; population genetics

We confirm that we have read this submission and believe that we have an appropriate level of expertise to confirm that it is of an acceptable scientific standard.

# Reviewer report: Reviewer response for version 1

**DOI:** 10.21956/wellcomeopenres.21609.r60901

**Published:** 2023-08-17

**Authors:** Shannon M Hedtke

**Affiliations:** 1 Department of Environment and Genetics, School of Agriculture, Biomedicine and Environment, La Trobe University, Melbourne, Victoria, Australia

This is a high-quality assembly of a kleptoparasitic bee, *Coelioxys conoidea*. I have no criticisms of the methods or results as the manuscript details well-accepted practices for genome assembly.

 The paper is well-written and the methods are easy to follow. I have a couple very minor comments for the authors to consider:

1. The justification for sequencing this organism in the text was limited to the project being part of the Darwin Tree of Life project, but what makes this genome exciting is its potential contribution to research in brood parasitism, which has evolved multiple times in Apoidea. I would encourage the authors to mention this in the last paragraph of the background.
2. Please specify lat-lon when reporting the coordinates in the beginning of the genome sequence report section.

Are sufficient details of methods and materials provided to allow replication by others?

Yes

Is the rationale for creating the dataset(s) clearly described?

Yes

Are the datasets clearly presented in a useable and accessible format?

Yes

Are the protocols appropriate and is the work technically sound?

Yes

Reviewer Expertise:

Ecological genomics; parasitology; entomology; phylogenetics; population genetics

I confirm that I have read this submission and believe that I have an appropriate level of expertise to confirm that it is of an acceptable scientific standard.
